# Towards sweetness classification of orange cultivars using short-wave NIR spectroscopy

**DOI:** 10.1038/s41598-022-27297-2

**Published:** 2023-01-06

**Authors:** Ayesha Zeb, Waqar Shahid Qureshi, Abdul Ghafoor, Amanullah Malik, Muhammad Imran, Alina Mirza, Mohsin Islam Tiwana, Eisa Alanazi

**Affiliations:** 1 Department of Electrical Engineering, Military College of Signals, National University of Sciences and Technology, Rawalpindi, 46000 Pakistan; 2 Robot Design and Development Lab. National Centre of Robotics and Automation, College of Electrical and Mechanical Engineering, National University of Sciences and Technology, Rawalpindi, 46000 Pakistan; 3 Institute of Horticultural Sciences, University of Agriculture, Faisalabad, Pakistan; 4 Department of Computer Science, Umm Al-Qura University, Mecca, Saudi Arabia; 5 School of Computer Science, Technological University Dublin, Dublin, D07 H6K8 Ireland

**Keywords:** Imaging and sensing, Electrical and electronic engineering

## Abstract

The global orange industry constantly faces new technical challenges to meet consumer demands for quality fruits. Instead of traditional subjective fruit quality assessment methods, the interest in the horticulture industry has increased in objective, quantitative, and non-destructive assessment methods. Oranges have a thick peel which makes their non-destructive quality assessment challenging. This paper evaluates the potential of short-wave NIR spectroscopy and direct sweetness classification approach for Pakistani cultivars of orange, i.e., Red-Blood, Mosambi, and Succari. The correlation between quality indices, i.e., Brix, titratable acidity (TA), Brix: TA and BrimA (Brix minus acids), sensory assessment of the fruit, and short-wave NIR spectra, is analysed. Mix cultivar oranges are classified as sweet, mixed, and acidic based on short-wave NIR spectra. Short-wave NIR spectral data were obtained using the industry standard F-750 fruit quality meter (310–1100 nm). Reference Brix and TA measurements were taken using standard destructive testing methods. Reference taste labels i.e., sweet, mix, and acidic, were acquired through sensory evaluation of samples. For indirect fruit classification, partial least squares regression models were developed for Brix, TA, Brix: TA, and BrimA estimation with a correlation coefficient of 0.57, 0.73, 0.66, and 0.55, respectively, on independent test data. The ensemble classifier achieved 81.03% accuracy for three classes (sweet, mixed, and acidic) classification on independent test data for direct fruit classification. A good correlation between NIR spectra and sensory assessment is observed as compared to quality indices. A direct classification approach is more suitable for a machine-learning-based orange sweetness classification using NIR spectroscopy than the estimation of quality indices.

## Introduction

Oranges are juicy, refreshing, and Pakistan’s most loved winter fruit. Pakistan is the 6th largest producer of citrus in the world^1^, and around 0.46 million tons of fruit were exported in the year 2020^2^. Ripeness is very critical as it directly influences the eating quality of harvested fruits^3^. Oranges are non-climacteric fruits i.e., they don’t ripe further once they are harvested. In Pakistan, quality inspection for fruits to be exported is still carried out subjectively by the packaging industry using sample-based tasting and/or by visualizing physical features, such as fruit color and size. The method is error-prone and tedious. These factors serve as a motivation for the automation of testing procedures. To automate the visual quality inspection, one can utilize camera sensors for estimating size, surface characteristics, and texture^4^. For gauging taste, sweetness, or other quality measures, one can utilize near-infrared (NIR) spectroscopy-based methods^5^. The non-destructive assessment using NIR spectroscopy can help to correlate dry matter (DM), Brix, titratable acidity (TA), and colour^6^ with fruit quality. Such assessment can also help in full batch testing and quality-based segregation as opposed to sample-based manual judgment.

Over the past decades, NIR spectroscopy has gained considerable attention for non-destructive maturity index assessment due to its ease, fast detection speed, and precision^7,8^. Researchers have used NIR spectroscopy with machine learning regression algorithms to develop maturity index prediction models such as DM, Brix, color, chlorophyll, starch and TA (only in high acid fruit like lemon and mandarin) of various fruits including apple^9^, pear^10^, nectarine^11^, mango^12^, banana^13^, melon^14^, mandarin^15^, strawberry^16^, apricot^17^, kiwifruit^18^, carambola^19^, grape^20^, loquat^21^ and pineapple^22^. However, due to the diversity in varieties and growing conditions, it is essential to develop the maturity index prediction model for a particular variety, growing region, and for local or export varieties^23^. Other applications require direct classification by use of some machine learning classification algorithm rather than quantification of quality parameter levels. For example, nectarine cultivars^24,25^, orange cultivars^26^, and orange growing regions^27^ have been differentiated, maturity classes of durian^28^, avocado^29^ and mango fruit^30^, internal defects detection of mango^31^, citrus^32^ and apple^33^, storage potential classification of kiwi^34^ and sweetness levels of melon^35^ and grapes^36^ have been classified.

Most of the published research on the measurement of intact fruit internal parameters have used wider wavelength regions including extended NIR region (> 1000 nm)^7^, e.g. for ‘Valencia’ orange 450–2500 nm^37^, for citrus 1100–2500 nm^38^, for ‘Satsuma’ mandarin 400–2350 nm^39^. The short-wave NIR (SWNIR) region (750–1100 nm) is used commercially for the assessment of internal quality attributes of intact fruit, in preference to the extended NIR region^7^. Longer wavelength ranges offer narrower and stronger absorption features as compared to SWNIR and thus better evaluation of internal parameters however, the SWNIR wavelengths have greater effective penetration depth into the fruit, hence, offer robustness across independent populations and given the variation in outer layer attributes. The short-wave Vis–NIR option is preferred for commercial purposes due to (currently) lower hardware costs^7,8^. Kim et al.^40^ reported RMSE_P_ and R_P_ of 0.514 ^o^Brix and 0.80 respectively for ‘Unshiu’ orange Brix prediction PLSR model trained using wavelength range 472-1156 nm. Luo et al.^41^ for Brix prediction of ‘Navel’ orange built PLSR model using wavelength range 450–1000 nm and reported RMSE_P_ and R_P_ of 1.35 ^o^Brix and 0.80, respectively. It is observed in both of the experiments^40,41^ that the wavelength region includes visible region as well along with SWNIR region and the results are for a single cultivar dataset. McGlone et al.^42^ used NIR direct transmission measurement mode with a spectral window of 700–930 nm for Brix and TA prediction of ‘Satsuma’ mandarin. The best results for Brix prediction are R and RMSE_P_ of 0.96, 0.32% and for TA it is stated that accurate TA prediction was not possible.

The pulp of oranges is covered inside a thick peel, which makes penetration of NIR spectroscopy challenging. Since ripening and harvest maturity is the same for non-climacteric fruits, there can be two ways to estimate ripeness/maturity. The first method is to estimate the fruit quality parameters like Brix, TA, etc. using a machine learning regression algorithm and based on their values judge the sample quality as done in^15,37–42^. The second method is to directly classify the eating quality using a machine learning classification algorithm, as reported by researchers in^35,36^ for the direct sweetness classification of melons and grapes. To the best of author’s knowledge, SWNIR spectroscopy is never investigated for direct sweetness classification of orange fruit. Moreover, the potential of SWNIR spectroscopy and direct sweetness classification for mixed cultivar datasets needs to be analyzed.

Like oranges, melons also have a thick rind. Zeb et al.^35^ have previously proposed a direct sweetness classifier for melons as opposed to Brix-based thresholding, using the correlation between short-wave NIR spectroscopy and sensory assessment. The proposed direct sweetness classifier tested on a single cultivar of melons i.e., ‘honey’ melons, outperformed the Brix estimation-based indirect classification method^35^. There is a need to evaluate the correlation of SWNIR spectroscopy and sensory assessment in other fruits as well and mixed cultivar datasets. As an extension of the Zeb et al.^35^ work, in this paper, the potential of SWNIR spectroscopy and direct sweetness classification through machine learning modelling is evaluated for Pakistani cultivars of orange i.e., Blood red, Mosambi, and Succari (average peel thickness 6 mm). A correlation is developed between quality indices i.e., Brix, TA, Brix: TA, and BrimA (Brix minus acids), the sweetness of the fruit, and NIR spectra which are then classified as sweet, mixed, and acidic using a machine learning classifier based on NIR spectra. We argue that direct classification is more suitable to evaluate orange sweetness as opposed to estimating quality indices.

## Materials and methods

### Fruit samples

Orange (*Citrus sinenses* (L.) Osbeck), cultivars (cvs.) ‘Blood red’, ‘Mosambi’ and ‘Succari’) ripened samples were harvested with due permission from orchard (Ghulam Rasool Farms) located in Chakwal district of Punjab province on two dates i.e., the first one started on 10th Jan 2021 and the second one on 28th Jan 2021 (33 of Blood red, 32 of Mosambi and 27 of Succari; 92 fruits in total). Average peel thickness was 6 mm. Sixty-four samples were used for model calibration, with each fruit scanned on two sides for Brix and TA to give 128 spectra. Twenty-eight samples (total 56 spectra) were used for model validation (see Table 1 for details). Samples within each fruit were treated as independent spectral set. All methods related to sample collection, destructive testing and sensory assessment were performed in accordance with the relevant guidelines/regulations/legislation.

**Table 1:** Number of samples of investigated orange cultivars in calibration and prediction datasets.

Cultivar	Number of samples in calibration set	Number of samples in prediction set
Blood red	23	10
Mosambi	22	10
Succari	19	8
Total	64	28

### Collection of Vis/NIR spectra

**Figure 1:**
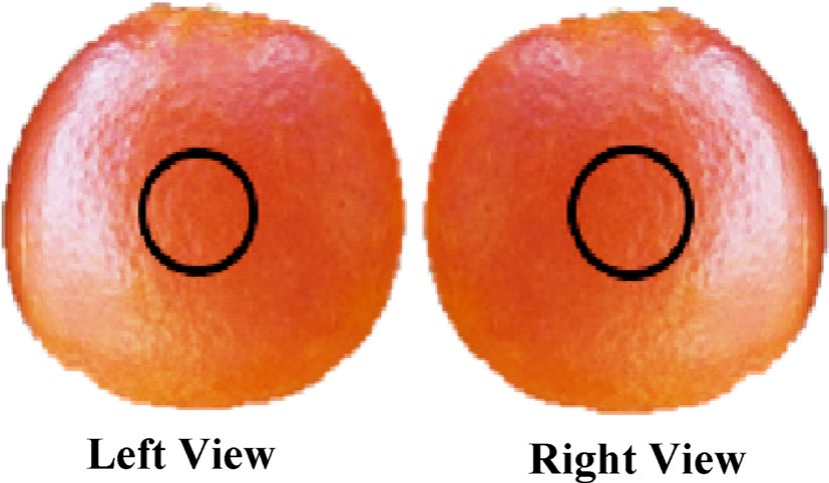
Schematic diagram of the marked positions for NIR spectra collection in oranges.

Orange samples were marked on-tree on opposite sides i.e. sun facing side and non-sun facing side (180° apart approximately) as shown in Fig. 1, to account for within fruit variations. After marking samples on-tree, the oranges were harvested on two dates (both harvest dates were one week apart) and brought to a local laboratory at National Centre of Robotics and Automation (Islamabad, Pakistan) and stored at room temperature for 24 h to minimize the influence of sample temperature on prediction accuracy^43^. Three spectra were collected from each position and average was computed. Vis–NIR spectra (range 400–1150 nm) were collected using the F-750 (Felix Instruments, Camas, WA, USA). This device employs interactance optical geometry and a Carl Zeiss MMS-1 spectrometer, with a pixel spacing of approximately 3.3 nm and a spectral resolution (FWHM) of 8–13 nm. It uses a halogen lamp as a light source.

### Reference measurements

For reference measurements, the marked region (along with surrounding tissues to get a suitable representation of the core as well) was excised and skin was removed. The extracted flesh was squeezed using a garlic press. Brix was assessed of a sample of the extracted juice using a digital refractometer (Model: PAL-1 [°Brix 0–53%], Atago Co., Ltd, Tokyo, Japan). The refractometer has automatic temperature compensation with range 10–100 °C and measurement accuracy of ± 0.2%.

TA was measured by manual titration of 10 mL of extracted juice with 0.1 M sodium hydroxide (NaOH) using phenolphthalein as an indicator. The acid formula for citrus fruit samples (Eq. 1) was applied to calculate TA, expressed as % citric acid.

1 $$TA \left(\% citric \, acid\right)= \frac{0.0064*titre \left(NaOH\right)mL}{10mL (juice)} x 100$$

2 $$Brix \, to \, TA \, ratio \left(maturity \, index\right)= \frac{Brix}{TA}$$

3 $$BrimA=Brix-k(TA)$$

Maturity index and BrimA were then calculated by Eqs. (2) and (3) respectively. The value of k in Eq. (3) is taken as 1.

### Sensory assessment

Reference values for sweetness were assessed by a briefly trained five judges panel with age between 20 and 50. After spectra acquisition, two slices were cut from the neighbor region from where destructive testing has been performed and presented to two of the judges at random for taste evaluation. Distilled water was provided to judges for drinking after every sample evaluation to clear previous sample taste. Oranges were classified into three classes by sensory evaluation i.e. Sweet, mix (sweet and acidic both) and acidic. The class label of each sample was described by average score of the two judges for that sample. Class wise scoring sheet used for assessment is given in Table 2.

**Table 2:** Score distribution for classification of sweetness level of oranges.

Class label	Score
Sweet	8–10
Mix	5–7
Acidic	0–4

### Chemometric analysis

**Figure 2:**
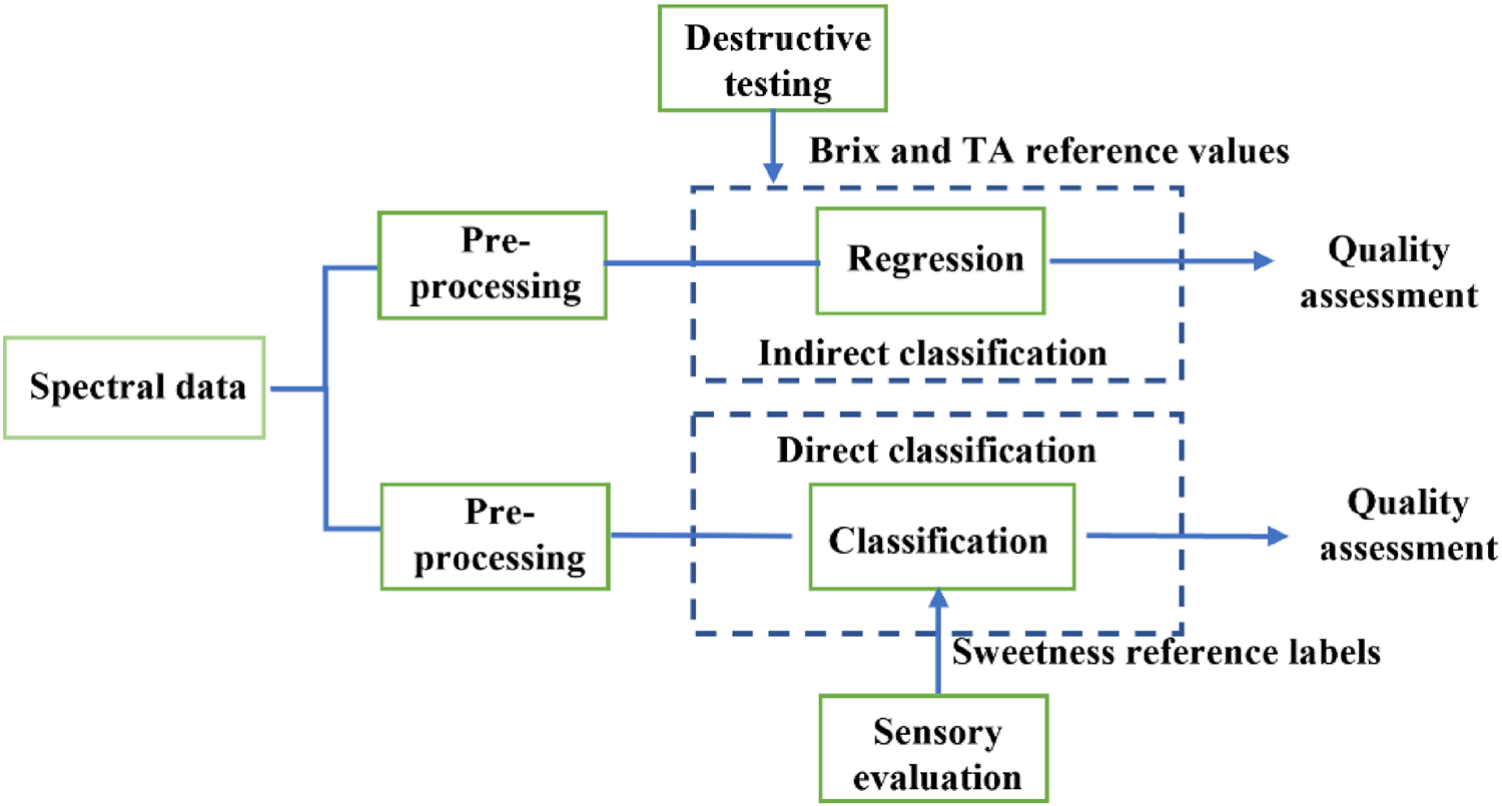
Block diagram representing two different methods of orange quality assessment.

A direct sweetness classification method has been proposed^35^ by authors for melons sweetness classification as opposed to indirect measure of Brix estimation. As an extension of author’s previous work^35^, in this paper, we have investigated potential of both the methods for quality assessments of mix cultivar oranges as shown in Fig. 2. The first method exploits the correlation between NIR spectra and fruit quality index parameters to estimate these parameters using machine learning regression algorithm and based on those predicted values, the quality of the sample is classified. The second method exploits the correlation between NIR spectra and sensory assessment to directly classify test sample as sweet, acidic or mix class sample, using machine learning classification algorithm.

Savitzky-Golay (SG) second derivative spectral pre-processing is a famous pre-processing method that usually outperforms other pre-processing methods for spectral data analysis^44^. Hence, 11-point SG second derivative preprocessing was performed on spectral data. Amongst all regression algorithms, the partial least squares regression is the most widely used regression algorithm for prediction of fruit quality index parameters^45^. For indirect quality assessment, partial least squares regression was used to build Brix, TA, Brix:TA ratio and BrimA estimation models.

Principle component analysis (PCA) has been widely used with spectroscopic data^45^ to emphasize variation and bring out strong patterns in the data set. For direct sweetness classification, after pre-processing, PCA was applied on spectral data and then several supervised and unsupervised learning classifiers are implemented and compared including tree, ensemble, K nearest neighbor (KNN), linear discriminant analysis (LDA) and SVM.

For indirect classification, the Unscrambler v11.0 spectral analysis software evaluation version (CAMO PRECESS AS, Oslo, Norway) was used for building combined variety calibration model using calibration dataset (Table 4). 11 points Savitzky-Golay second derivative smoothing filter was applied before building model. The performance of developed models was evaluated by R_CV_ (correlation coefficient of cross validation), R_P_ (correlation coefficient of prediction), RMSE_CV_ (root mean square error of cross validation) and RMSE_P_ (root mean square error of prediction). Tenfold cross validation was performed. Prediction models were developed using the Vis/NIR region in the range 600–1050 nm (following^41^).

For direct classification, MATLAB R 2018a software was used. Input data for both the methods i.e. direct and indirect classification was same (600–1050 nm wavelength values pre-processed with 11-point SG second derivative using Unscrambler software). Classification was performed using MATLAB classification learner module with PCA enabled (first 15 principal components were used).

## Results

### Dataset statistics

Destructive testing statistics of orange quality index parameters i.e. Brix, TA, maturity index and BrimA with respect to the individual variety are shown in Table 3. The range and mean of Blood red cultivar is relatively low for Brix, Brix:TA ratio and BrimA, and high for TA as compared to other two varieties. Table 3 shows that the statistics of Succari cultivar are dissimilar from the other two investigated cultivars with respect to TA and Brix:TA ratio i.e. TA range (0.14–0.33%) and mean (0.21%) is lowest and maturity index range (33.64–75.63) and mean (55.38) is highest than that of Blood red and Mosambi cultivars.

**Table 3:** Statistics of Brix, TA, maturity index and BrimA with respect to the individual investigated varieties of orange.

Dataset	Number of samples	Range				Mean				S.D			
		Brix (^o^Brix)	TA (%)	Brix:TA ratio	BrimA (%)	Brix (^o^Brix)	TA (%)	Brix:TA ratio	BrimA (%)	Brix (^o^Brix)	TA (%)	Brix:TA ratio	BrimA (%)
Blood red	33	7.3–11.3	0.59–1.98	5.3–12.71	6.53–9.97	9.22	1.03	9.37	8.2	1.04	0.29	1.67	0.88
Mosambi	32	9–13.4	0.4–1.12	9.82–24.69	8.51–12.73	10.98	0.68	16.9	10.31	1.22	0.19	3.57	1.12
Succari	27	8.8–13.1	0.14–0.33	33.64–75.63	8.54–12.9	11.03	0.21	55.38	10.77	1.02	0.04	11.09	0.99

Since, Succari cultivar is statistically different from the other two cultivars, the models were built using two different combinations of investigated cultivars i.e. dataset-1 contains all three investigated cultivars and dataset-2 contains only Blood red and Mosambi cultivars. Table 4 shows data set wise statistics of quality index parameters.

**Table 4:** Statistics of reference values with respect to calibration and prediction data sets.

Dataset		Total samples	Min				Mean				S.D			
			Brix (^o^Brix)	TA (%)	Brix:TA ratio	BrimA (%)	Brix (^o^Brix)	TA (%)	Brix:TA ratio	BrimA (%)	Brix (^o^Brix)	TA (%)	Brix:TA ratio	BrimA (%)
Dataset1: (Blood red, Mosambi and Succari)	Calibration	128	7.4–13.4	0.14–1.98	5.3–75.63	6.53–12.73	10.37	0.69	24.91	9.68	1.39	0.39	20.89	1.49
	Prediction	56	7.3–13.1	0.17–1.5	6.2–65.5	6.56–12.9	10.3	0.62	25.87	9.64	1.36	0.37	19.70	1.55
	Total	184	7.3–13.4	0.14–1.98	5.3–75.63	6.53–12.9	10.36	0.67	25.20	9.70	1.39	0.39	20.48	1.52
Dataset2: (Blood red and Mosambi)	Calibration	90	7.4–13.4	0.4–1.98	5.3–22.94	6.53–12.73	10.20	0.89	12.54	9.31	1.52	0.29	4.22	1.53
	Prediction	40	7.3–12.1	0.4–1.5	6.2–24.69	6.56–11.61	9.83	0.79	14.17	9.03	1.20	0.30	5.44	1.25
	Total	130	7.3–13.4	0.4–1.98	5.3–24.69	6.53–12.73	10.08	0.86	13.05	9.23	1.43	0.3	4.67	1.45

**Figure 3:**
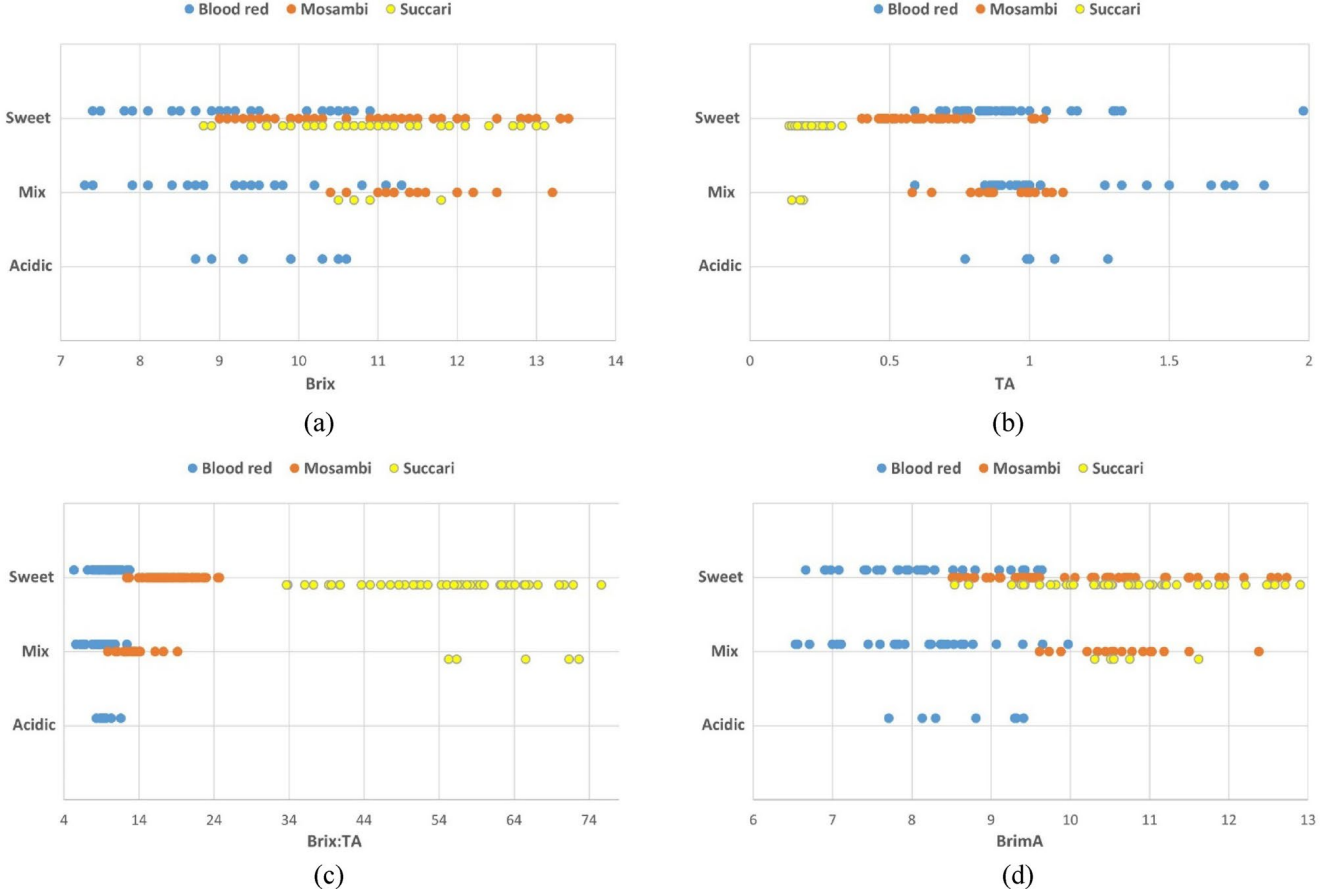
Distribution of (**a**) Brix, (**b**) TA, (**c**) maturity index and (**d**) BrimA with respect to orange taste quality levels.

Figure 3 shows the distribution of quality index values with respect to orange sweetness levels. From 184 samples (92 oranges, 2 samples each), 129 samples belonged to sweet class, 48 belonged to mix class and 7 belonged to acidic class. From Fig. 3, the sweetness levels cannot be concluded based on individual values of Brix, TA, Brix:TA or BrimA, since there is significant overlap between the three sweetness levels and the respective quality indexes. Moreover, it can be concluded that with respect to quality index parameters, Succari cultivar is dissimilar to the other two investigated varieties.

**Figure 4:**
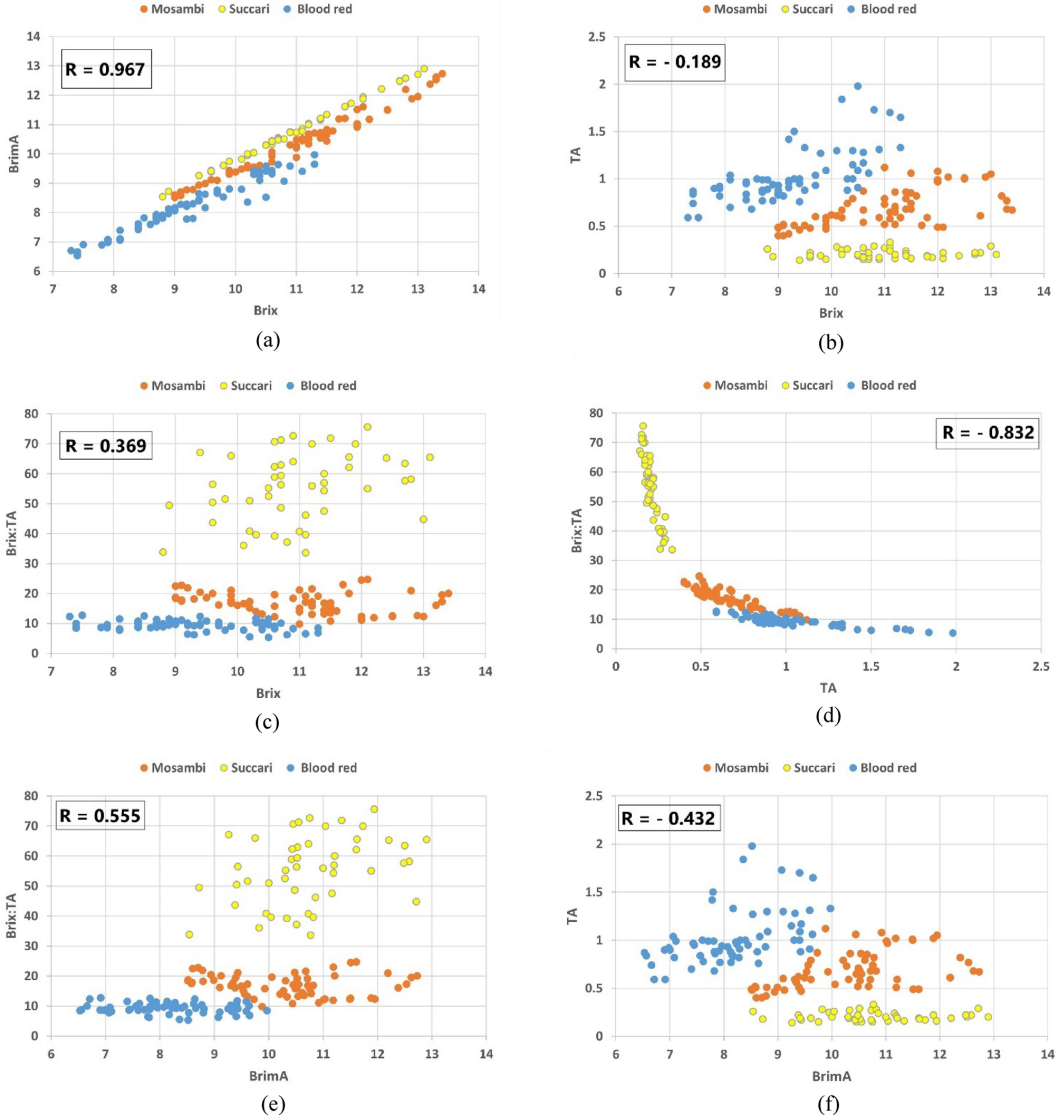
Scatter plots depicting statistical correlation among (**a**) Brix vs BrimA (**b**) Brix vs TA, (**c**) Brix vs maturity index, (**d**) TA vs maturity index, (**e**) BrimA vs maturity index, and (**f**) BrimA vs TA for all three cultivars.

Figure 4 and Table 5 show the statistical correlation amongst the quality indices. Brix and BrimA (Fig. 4a) show a strong positive correlation (R = 0.967) while TA and maturity index (Fig. 4d) show negative correlation (R = − 0.832) for all the three investigated cultivars. Other scatter plots (Fig. 4b,c,e,f) do not show a strong positive/negative correlation amongst the indices for all the investigated cultivars. Table 5 shows that there is no correlation between Brix vs TA and BrimA vs TA for Succari samples.

**Table 5:** Correlation coefficient (R) values among quality parameters with respect to individual cultivars and combined dataset.

Parameters	Correlation coefficient (R)			
	Blood red	Mosambi	Succari	All three cultivars
Brix vs BrimA	0.971	0.991	0.999	0.967
Brix vs TA	0.681	0.613	0.087	− 0.189
Brix vs maturity index	− 0.336	− 0.268	0.320	0.369
BrimA vs maturity index	− 0.114	− 0.139	0.360	0.555
TA vs Maturity index	− 0.877	− 0.898	− 0.893	− 0.832
BrimA vs TA	0.485	0.5	0.043	− 0.432

### Overview of spectra

**Figure 5:**
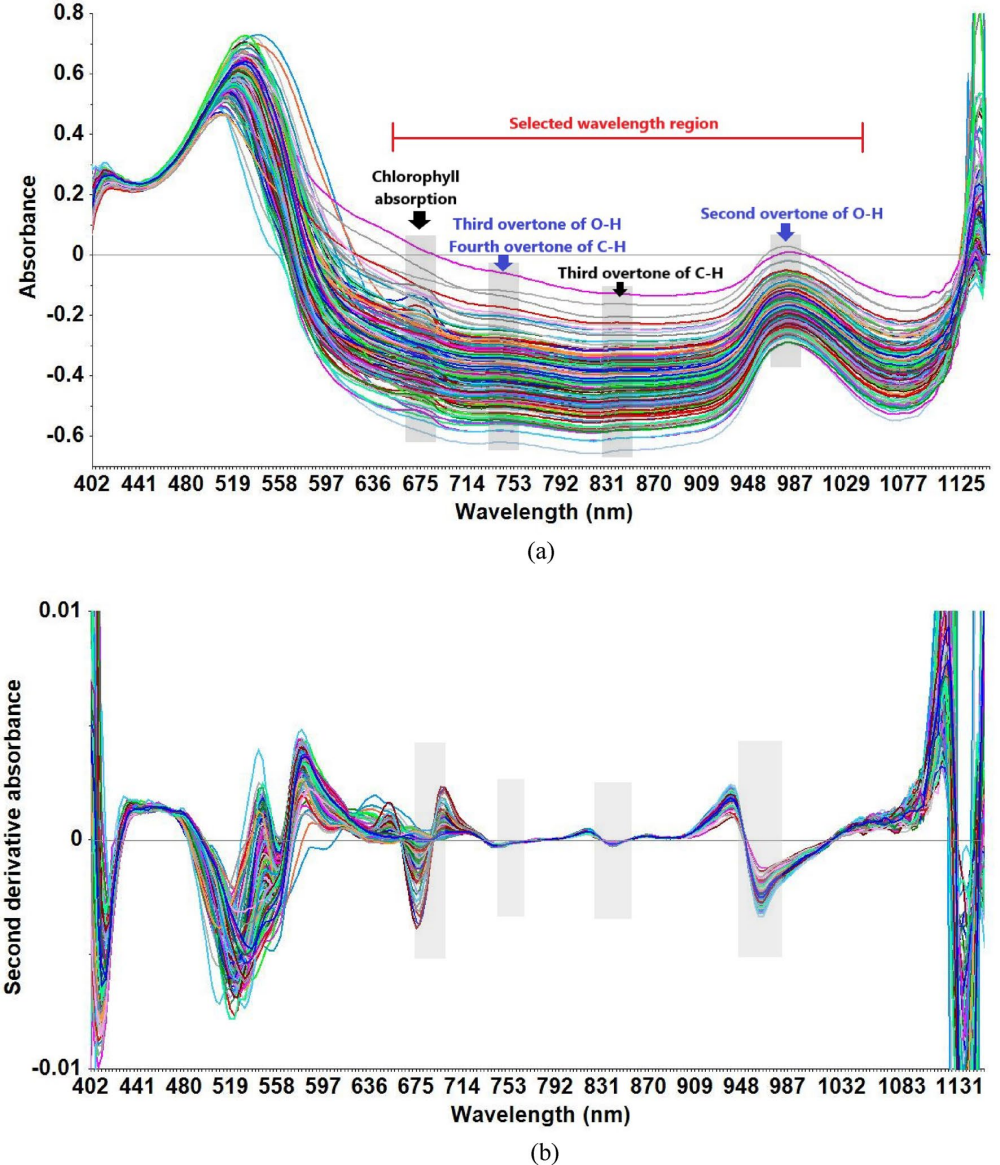
(**a**) Raw absorbance and (**b**) Savitzky–Golay second derivative spectra of collected dataset.

The absorbance spectra of orange fruit (Fig. 5a) is dominated by a peak around 680 nm associated to chlorophyll absorption^46^. Moreover, broad peaks around 750 nm and 850 nm are observed due to the third overtone of O–H bond stretching and the third and fourth overtones of C-H bond stretching^47^. Another observed peak at 970 nm is associated with water absorption band (second overtone of O–H bond stretching)^48^. Second derivative of the spectrum shown in Fig. 5b confirmed all above absorbance peaks. Prediction models were developed using the Vis/NIR region in the range 600–1050 nm (following^41^) as this is the region of carbohydrates such as glucose, fructose and sucrose^47^.

### Indirect classification results

Table 6 presents the combined variety PLSR model results on Brix, TA, Brix:TA and BrimA with dataset having 184 samples including all three investigated varieties. The cross validation R is 0.69, 0.48, 0.5 and 0.66 respectively and RMSE is 1.00 ^o^Brix, 0.34%, 18.06 and 1.13% respectively.

**Table 6:** Cross validation and prediction results for PLSR models developed for dataset1 (Blood red, Mosambi and Succari).

Index	PLSR model			
	Cross validation		Prediction	
	R_cv_	RMSE_cv_ (^o^Brix/%)	R_P_	RMSE_P_ (^o^Brix/%)
Brix (^o^Brix)	0.69	1.00	0.57	1.05
TA (% citric acid)	0.48	0.34	0.25	0.48
Brix:TA ratio	0.5	18.06	0.39	20.99
BrimA (%)	0.66	1.13	0.55	1.35

**Figure 6:**
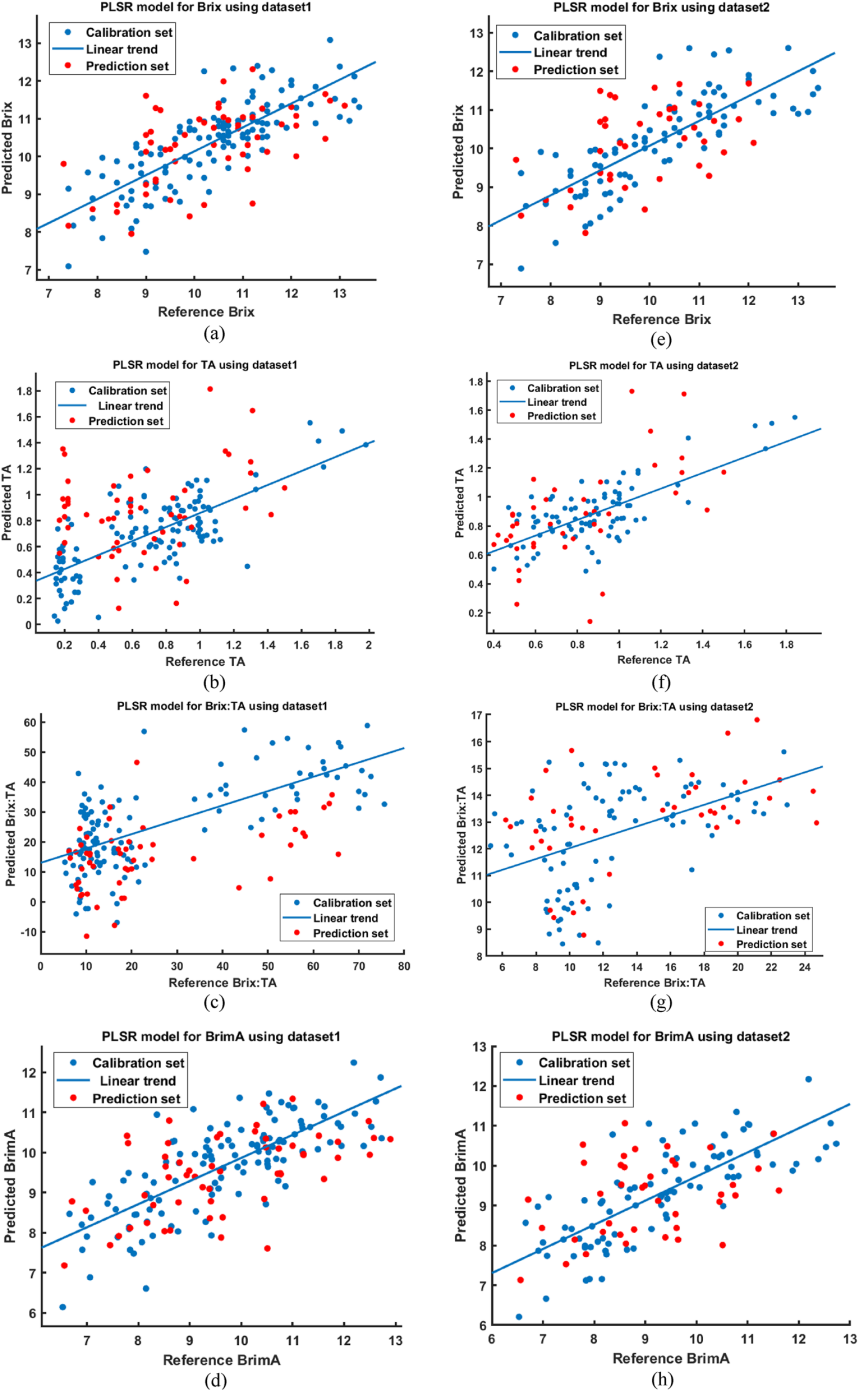
Scatter plots of predicted vs reference values for PLSR models using dataset 1 (**a**) Brix, (**b**) TA, (**c**) Brix:TA and (**d**) BrimA, and dataset 2 (**e**) Brix, (**f**) TA, (**g**) Brix:TA and (**h**) BrimA.

These models include samples of Succari variety as well, which is a statistically incompatible cultivar (with respect to TA and Brix:TA) with the Blood red and Mosambi cultivars. Hence, Table 7 shows PLSR models trained on Blood red and Mosambi cultivars since they are similar to each other w.r.t TA and Brix:TA statistics. Table 6 shows that excluding Succari samples from dataset and rebuilding PLSR models provided improved results for TA and Brix:TA models. However, Brix and BrimA prediction results were worsened because with respect to Brix, all three investigated varieties have similar statistics. Removing Succari samples reduced the size of data set and hence worse results. Figure 6 shows the scatter plots of predicted vs reference values of the developed PLSR models of Tables 6 and 7.

**Table 7:** Cross validation and prediction results for PLSR models developed for dataset2 (Blood red and Mosambi).

Index	PLSR model			
	Cross validation		Prediction	
	R_cv_	RMSE_cv_ (^o^Brix/%)	R_P_	RMSE_P_ (^o^Brix/%)
Brix (^o^Brix)	0.83	1.10	0.43	1.18
TA (% citric acid)	0.59	0.23	0.73	0.19
Brix:TA ratio	0.43	3.79	0.66	3.14
BrimA (%)	0.58	1.23	0.29	1.33

### Direct classification results

To predict orange’s eating quality in terms of sweetness, multi class classification algorithms were implemented on both datasets. The cross validation and prediction result for both data sets are listed in Tables 8 and 9. For dataset1, ensemble classifier achieved 81.03% accuracy for 3 class classification of independent test data. For dataset2, SVM and KNN both achieved 79.49% accuracy for 3 class classification of independent test data.

**Table 8:** Cross validation and prediction results for 3 class classification for dataset1 (Blood red, Mosambi and Succari cultivars).

Classifiers	Cross validation accuracy (%)	Prediction set accuracy (%)
Tree	57.5	72.41
LDA	56.7	60.34
SVM	64.2	60.34
KNN	63.4	72.41
Ensemble	58.2	**81.03**

Significant values are given in bold.

**Table 9:** Cross validation and prediction results for 3 class classification for dataset2 (Blood red and Mosambi cultivars).

Classifiers	Cross validation accuracy (%)	Prediction set accuracy (%)
Tree	57.8	64.10
LDA	53.3	76.92
SVM	60	**79.49**
KNN	66.7	**79.49**
Ensemble	57.5	71.79

Significant values are given in bold.

## Observations and discussion

### Statistics comparison of investigated cultivars

The “Blood red” variety is the most tasteful (mix to sweet taste) cultivar of orange in Pakistan. Table 3 shows that the range and mean of TA are high and of Brix, Brix: TA, and BrimA are low. Of 66 samples of Blood red, 33 belonged to sweet class, 26 belonged to mix class, and 7 belonged to acidic class.

The Mosambi cultivar is also segregated as sweet by the judges. It can be seen from Table 3 that its range and mean of TA are lesser and for Brix, it’s higher than the Blood red cultivar hence its flavor is generally more sweater than Blood red variety. Amongst 64 samples of Mosambi, 46 belonged to the sweet class and 17 belonged to the mixed class.

The Succari cultivar is a different cultivar in terms of sweetness from the other two cultivars. Succari samples always have a flat sweet taste due to a lack of acid contents. The statistics of quality index parameters also support this claim as its TA range and mean is the lowest and hence Brix: TA values are the highest amongst other investigated. Amongst 54 samples of Succari, 47 belonged to the sweet class and only 5 belonged to the mixed class.

### Development of mixed cultivar PLSR models

An attempt was made to predict Brix, TA, Brix: TA, and BrimA using PLSR regression models developed for mixed cultivar datasets. Since the Succari cultivar is statistically (w.r.t TA and Brix: TA) and taste-wise different from the other two investigated cultivars, PLSR models were built for two datasets, one having a mixture of statistically different cultivars i.e. Blood red, Mosambi and Succari and other one having only statistically compatible cultivars i.e. Blood red and Mosambi.

It is observed that since all three investigated cultivars have almost similar Brix and BrimA statistics and are positively correlated (Table 3 and Fig. 4a), hence the model built with data set having all three cultivars achieved better prediction results for Brix and BrimA as compared to the model built with a dataset having only two cultivars i.e. Blood red and Mosambi (Tables 6, 7). This is because dataset 2 has a lesser number of samples than dataset1. The TA and Brix: TA results of PLSR models built with only two cultivars’ data (Blood red and Mosambi) achieved relatively better prediction results than the three cultivar dataset.

Dataset standard deviation (S.D.) is important to determine the value of the NIR spectroscopy technique for fruit quality assessment^7^. The technique holds significance only when the S.D. of the attribute of interest is greater than the measurement RMSE_P_. Indeed, the prediction set R is directly related to measurement bias corrected RMSEP and S.D. i.e., for a particular bias corrected RMSE_P_, higher S.D. will result in a higher R_P_ value^7^.

For indirect classification, it is observed that the R_CV_ and R_P_ values of the developed PLSR models are low however, the RMSE_CV_ and RMSE_P_ are below the S.D. of the datasets (for Brix and BrimA considering S.D. of dataset1 and for TA and Brix: TA considering S.D. of dataset2) (see Tables 3, 4, 5, 6 and 7). The low R_P_ values are because of the low S.D. of the collected dataset, which is a limitation for the presented work as well. Due to the low R_CV_ and R_P_ values, estimation of quality index value using PLSR models is not a suitable option with the investigated dataset, rather the overall sorting using the classification of sweetness levels is a suitable option.

We observed (see Tables 8 and 9) a good correlation between NIR spectra and sensory assessment as opposed to quality indices. Hence, like melons^35^, direct classification is more suitable for mixed cultivar orange sweetness classification using NIR spectroscopy as opposed to the estimation of quality indices.

## Conclusion

The research was carried out to investigate the correlation between quality indices i.e. Brix, titratable acidity (TA), Brix: TA, and BrimA (Brix minus acids), sensory assessment of the fruit, and short wave near-infrared (SWNIR) spectra that were then classified as sweet, mixed, and acidic based on SWNIR spectra for mixed cultivar datasets. Datasets were collected using three Pakistani cultivars of orange i.e., Blood red, Mosambi, and Succari cultivars. It is observed that Succari cultivar is a statistically different cultivar (w.r.t. TA and Brix:TA values) than Blood red and Mosambi cultivars. Hence, two experiments were performed: one with samples of Blood red, Mosambi, and Succari (dataset1), the second with samples of statistically similar cultivars (dataset2) i.e., Blood red and Mosambi. Given both the datasets, the best fit PLSR model for Brix and BrimA is obtained with dataset1 while for TA and Brix:TA, the best fit model is obtained with dataset2. It is concluded that to develop a statistical model, samples of statistically dissimilar cultivars should not be merged to form a single mixed cultivar dataset. Moreover, we observed a good correlation between SWNIR spectra and sensory assessment as opposed to quality indices. Hence, direct classification machine learning model is more suitable for orange sweetness classification using SWNIR spectroscopy as opposed to the developing a machine learning model for estimation of quality indices (Supplementary information S1).

## Supplementary Information

Supplementary Information.

## Data Availability

The data that support the findings of this study and/or analyzed during the current study available from the corresponding author on reasonable request.
